# Cast Removal by Soaking Reduces Infant and Parental Anxiety Without Affecting Clinical Outcomes in Ponseti-Treated Idiopathic Clubfoot: A Prospective Controlled Trial

**DOI:** 10.3390/children13081007

**Published:** 2026-07-29

**Authors:** Tayfun Bacaksiz, Mehmet Maden, Meliksah Uzakgider, Serkan Erkus, Mirac Kadir Turhan, Ihsan Akan, Fatih Surenkok, Cemal Kazimoglu

**Affiliations:** 1 Department of Orthopaedics and Traumatology, Izmir Katip Celebi University Atatürk Training and Research Hospital, 35360 Izmir, Türkiye; tayfun_bacaksiz@hotmail.com (T.B.); drihsanakan@gmail.com (I.A.); ckazimoglu2000@yahoo.com (C.K.); 2 Department of Orthopaedics and Traumatology, Izmir City Hospital, 35540 Izmir, Türkiye; meliksahu@gmail.com; 3 Department of Orthopaedics and Traumatology, Izmir Economy University Medical Point Hospital, 35575 Izmir, Türkiye; s.erkus@ymail.com; 4 Department of Orthopaedics and Traumatology, Balıklıgol State Hospital, 63050 Sanliurfa, Türkiye; m_k_turhan@hotmail.com; 5 Department of Orthopaedics and Traumatology, Izmir Tınaztepe University Galen Hospital, 35001 Izmir, Türkiye; fatihsurenkok@dr.com

**Keywords:** idiopathic clubfoot, Ponseti method, cast removal, oscillating saw, soaking, complications

## Abstract

**Highlights:**

**What are the main findings?**

• Home-based cast removal by soaking was associated with clinical outcomes comparable to oscillating saw removal in infants treated with the Ponseti method. Second bullet.
• The soaking technique was associated with lower infant distress, reduced parental anxiety, and fewer cast-removal–related complications.

**What are the implications of the main finding?**

• Home cast removal by soaking may be considered a family-centered alternative to clinic-based oscillating saw removal for appropriately selected infants with idiopathic clubfoot.
• With standardized parental education, the soaking technique may improve the treatment experience without compromising treatment effectiveness.

**Abstract:**

**Background:** Cast removal is an essential but often stressful component of the Ponseti method for idiopathic clubfoot. The impact of different cast removal techniques on infant stress, parental anxiety, and treatment outcomes remains insufficiently explored. **Methods:** This prospective, quasi-randomized controlled trial included 84 infants with unilateral idiopathic clubfoot treated using the Ponseti method. Patients were allocated to cast removal by oscillating saw in the outpatient clinic (Group 1, n = 42) or by soaking and peeling at home prior to clinic visits (Group 2, n = 42). The primary outcome was the number of casts required to achieve correction and readiness for percutaneous Achilles tenotomy. Secondary outcomes included infant crying time, maximum heart rate during cast removal, parental anxiety assessed using the Short State Anxiety Inventory Scale (SAIS), cast removal time, hospital stay duration, and cast-removal–related complications. **Results:** There was no significant difference between groups regarding pre- or post-treatment Pirani and Dimeglio scores or the number of casts required (*p* > 0.05). Infant crying time, heart rate during cast removal, and parental anxiety scores were significantly higher in the oscillating saw group (*p* < 0.001). Cast-removal–related complications were more frequent in the oscillating saw group (*p* = 0.003). **Conclusions:** In this study, home-based soaking and peeling provided clinical outcomes comparable to oscillating saw removal and was associated with lower infant distress markers, lower parental anxiety scores, and fewer observed cast-removal–related complications. These findings suggest that soaking and peeling may represent a family-centered alternative for selected families receiving appropriate instruction.

## 1. Introduction

Idiopathic clubfoot is the most common congenital musculoskeletal deformity of the lower extremity. Early initiation of treatment with serial casting is widely accepted as the standard of care, with the Ponseti method demonstrating high rates of successful correction and long-term functional outcomes [1,2,3,4,5]. The technique involves sequential manipulations and above-knee casting, with percutaneous Achilles tenotomy required in the majority of cases to correct residual equinus deformity [1,4].

Plaster of Paris remains the most commonly used casting material in Ponseti treatment [6,7], although alternative synthetic and bio-based materials have been explored to optimize treatment efficiency and patient comfort [8,9,10]. As casts are replaced weekly, cast removal represents a repetitive and integral component of the treatment process. Several techniques are currently available for cast removal, including oscillating cast saws, manual removal using cast shears (Stille shears), and soaking with subsequent peeling of Plaster of Paris casts [11,12]. In routine practice, cast removal is most often performed in the hospital using oscillating saws. However, cast removal with oscillating saws has been associated with procedure-related complications such as skin abrasions and thermal injuries, as well as increased anxiety in pediatric patients and their caregivers [13].

Cast removal by soaking and peeling has been proposed as an alternative approach aimed at reducing procedure-related complications and improving family satisfaction [9,12]. Despite its potential advantages, this method is more time-consuming and requires a high level of parental compliance. While previous studies have compared different casting materials within the Ponseti method, data evaluating the impact of different cast removal techniques using the same casting material remain limited [8,9,14,15].

Manual cast removal with cast shears has been reported to be an effective method when performed by experienced clinicians, particularly in infants with clubfoot [11]. However, operator experience, availability of appropriate instruments, and institutional practice patterns influence the preferred removal technique. In our institution, oscillating saw removal and home soaking were the standard methods and therefore constituted the interventions evaluated in the present study.

The aim of this study was to compare oscillating saw removal and soaking-based cast removal techniques in infants treated with the Ponseti method, focusing on treatment outcomes, procedure-related complications, and anxiety levels in both patients and their parents.

## 2. Materials and Methods

This study was designed as a prospective, quasi-randomized controlled trial conducted after approval from the institutional ethics committee. The study was not prospectively registered in a publicly accessible clinical trial registry.

The primary outcome was the number of casts required to achieve correction. Secondary outcomes included anxiety-related parameters (crying time, heart rate, and parental SAIS score), procedural timing variables, and cast-removal–related complications.

Blinding of participants, caregivers, and outcome assessors was not feasible due to the nature of the interventions.

### 2.1. Patients

Infants diagnosed with unilateral idiopathic clubfoot within the first month of life were eligible. Written informed consent was obtained from all parents.

Exclusion criteria were determined as follows:

- Diagnosis of bilateral idiopathic clubfoot;
- Age > 1 month at presentation;
- Diagnosis of clubfoot associated with arthrogryposis, spina bifida, skeletal dysplasias, amniotic band syndrome or genetic syndromes;
- Diagnosis of positional clubfoot;
- Partially treated in an external center;
- Treatment was interrupted for any reason or lost to follow-up.

### 2.2. Allocation

Patients were allocated in a quasi-randomized alternating sequence based on outpatient clinic admission order. Eligible patients were consecutively assigned to Group 1 and Group 2 in an alternating manner according to the order of presentation to the clinic. This ensures an equal randomization independent of patients and researchers at a 1:1 ratio. However, due to the practical nature of the intervention, it was not possible to conceal group assignments.

### 2.3. Sample Size

**Figure 1:**
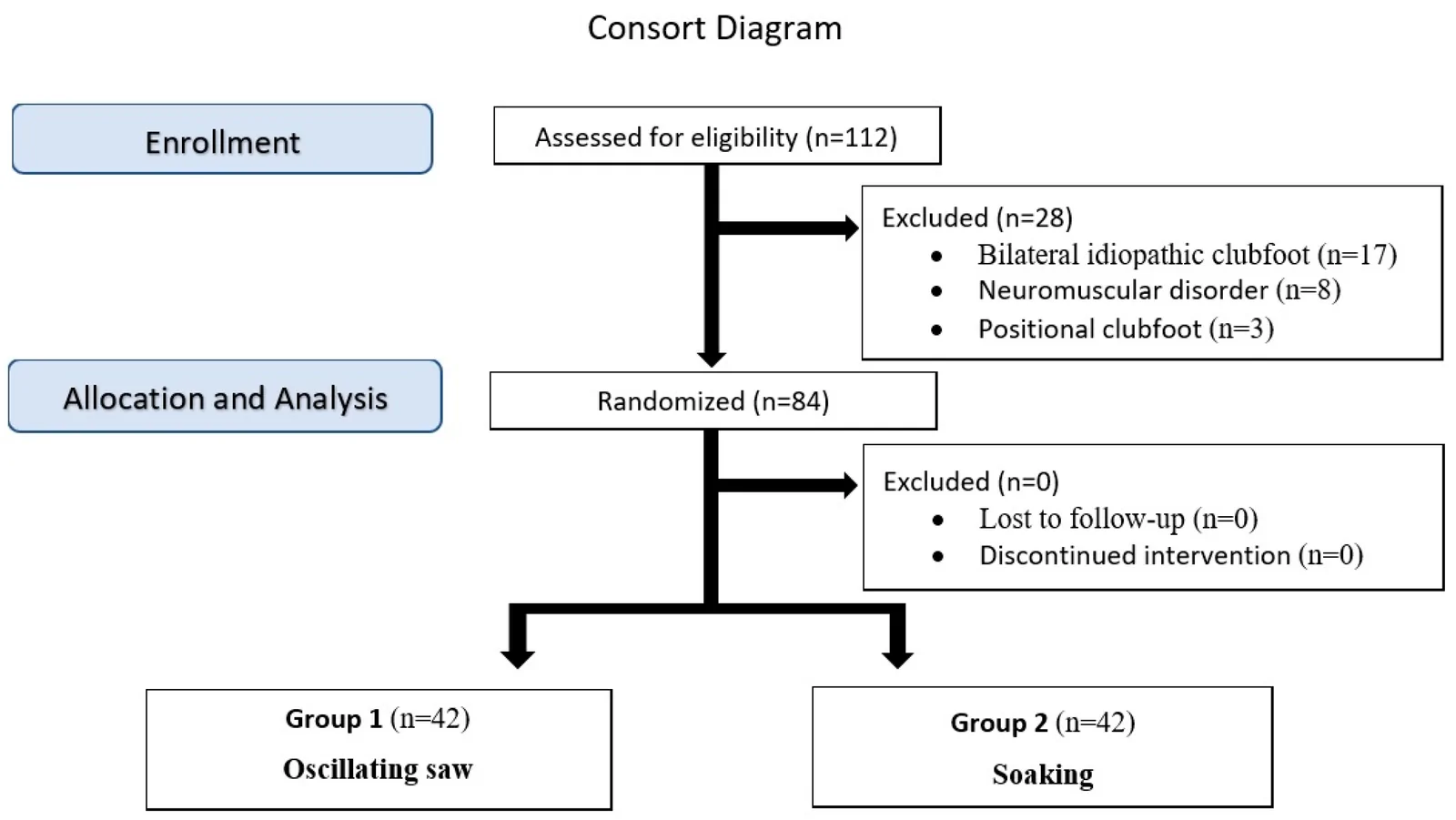
CONSORT flow diagram of patient enrollment and allocation.

The sample size was calculated using the number of casts required to achieve clinical success in the study in which the Ponseti technique was applied to the treatment of idiopathic clubfoot, with a 5% Type 1 error, an effect size of 0.57, a target of reaching 80% power, and a 10% loss margin by taking the group ratio as 1:1 in G*Power (Ver. 3.9.1.6 © Franz-Faul-Germany) [9]. In the sample calculation (Student’s *t*-test—comparison of two means), it was found that reaching 42 feet for each treatment group was sufficient. Considering that there were 2 groups in our study, we aimed to reach 84 feet with idiopathic clubfoot deformity (Figure 1).

### 2.4. Interventions

All patients were treated using the standard Ponseti technique with Plaster of Paris (POP) above-knee casts applied by the same pediatric orthopedic team and serial manipulations were applied weekly. The identical casting material was used throughout the study in both groups to ensure that the cast removal technique was the only procedural variable under investigation. Group 1 underwent cast removal in the outpatient clinic using an oscillating saw by an experienced technician under physician supervision. During cast removal, protective strips and manual soft tissue stabilization were routinely used to safely separate the edges of the plaster. However, because of the narrow shape and molding characteristics of Ponseti casts, insertion of protective strips was technically limited in some regions, particularly around the forefoot and hallux. Group 2 underwent cast removal at home by soaking and peeling the cast prior to clinic visits. Parents in Group 2 received standardized verbal and practical instruction during the first cast application. The soaking and peeling technique was first explained verbally and subsequently demonstrated by an experienced orthopedic surgeon using a model cast. As per standard procedure used in the study, the patient is placed on a flat bed. For the soaking technique, approximately 250–500 mL of warm tap water (approximately 37–39 °C) was applied over the cast using a cup without immersion bathing [12]. Gauze or a soft towel was used to maintain repeated wetting of the cast surface until the plaster softened adequately. The soaking process generally required approximately 10–15 min depending on cast thickness. The plaster was then peeled starting from the folded distal edge created during cast application. After the cast is removed, the lower extremity is cleaned with warm water and prepared for plaster application. Parents were instructed to discontinue the procedure and contact the treating physician if they experienced difficulty removing the cast or if any skin injury occurred. The next Ponseti cast is performed in the outpatient intervention room.

### 2.5. Evaluation

The patients’ age and demographic data were recorded in the case follow-up form. The affected foot was photographed before and after plaster treatment and during orthosis application. Clinical success was defined as the correction of cavus, adductus and varus deformities and the achievement of adequate ankle dorsiflexion, in accordance with the standard Ponseti protocol. Therefore, the Pirani and Dimeglio scores were recorded immediately prior to each plaster application by the senior orthopedic surgeon responsible for the application, who had not been informed of the patient’s group allocation.

The primary outcome of this study was the number of casts required to achieve correction of the extra-articular components of idiopathic clubfoot and readiness for percutaneous Achilles tenotomy, or complete correction of all deformity components with at least 15 degrees of ankle dorsiflexion.

Secondary outcomes included treatment- and procedure-related parameters and anxiety-related surrogate markers. Treatment-related outcomes comprised the total duration of casting (weeks), cast removal time, time spent in the hospital during cast change visits, and mean Pirani and Dimeglio scores recorded at each cast replacement.

Anxiety-related outcomes were assessed using indirect and physiological surrogate measures. Infant distress was evaluated by recording the total crying time during cast removal and subsequent cast application. In Group 1, the maximum heart rate during cast removal was measured using a pulse oximeter (NPB-40; Nellcor Puritan Bennett, Pleasanton, CA, USA) by the same orthopedic surgeon who supervised cast removal throughout the study, thereby ensuring a standardized measurement procedure. For Group 2, parents were provided with the same pulse oximeter and received standardized verbal and practical instruction on heart rate measurement during the initial casting session to ensure consistency across study groups. The data obtained in Group 2 consisted of parent-recorded crying durations and pulse oximeter heart rate measurements during home cast removal, consistent with methodologies previously described in the literature [16,17].

Cast-removal–related complications, including skin abrasion, hyperemia, and superficial skin lesions, were recorded prospectively. Parental anxiety was assessed in both groups using the Short Anxiety Inventory Scale (SAIS) after the cast removal procedure was completed. In both groups, the SAIS assessment was conducted immediately before the next cast application procedure, based on the anxiety level during the cast removal process. Mean SAIS scores were calculated for each patient across the treatment period. Interruptions in routine healthcare practices, including vaccination or scheduled health checks related to casting, were documented as exploratory observations during follow-up.

### 2.6. Statistical Analysis

Statistical analyses were performed using SPSS version 22 (IBM Corp., Armonk, NY, USA). Data distribution was assessed using the Kolmogorov–Smirnov test. Descriptive statistics were presented as mean ± standard deviation for normally distributed variables and as median with minimum–maximum values for non-normally distributed variables. Categorical variables were expressed as frequencies and percentages.

Between-group comparisons for continuous variables were performed using the independent samples *t*-test. Within-group comparisons of pre- and post-treatment clinical scores were conducted using the paired t-test. Repeated-measures analysis of variance with post hoc Tukey correction was applied to evaluate changes in clinical scores across successive cast applications when normality assumptions were met.

All statistical tests were two-sided, and a *p*-value < 0.05 was considered statistically significant.

## 3. Results

A total of 84 infants with unilateral idiopathic clubfoot met the inclusion criteria and completed the study. All families allocated to the soaking-and-peeling group successfully completed home cast removal throughout the treatment period. No participant required conversion to oscillating saw-assisted removal or any additional hospital-based cast removal because of failure of the home peeling technique. No crossover occurred between groups during follow-up. Patients who discontinued treatment or were lost to follow-up were to be excluded from the study; however, following randomization, all enrolled patients completed treatment and were included in the final analysis (Figure 1). The mean age at initiation of treatment was 6.48 ± 2.57 days (range, 2–13 days). Of the patients, 45 (53.6%) were male and 39 (46.4%) were female. There were no significant differences between the groups regarding age, sex distribution, or side of involvement (Table 1).

**Figure 2:**
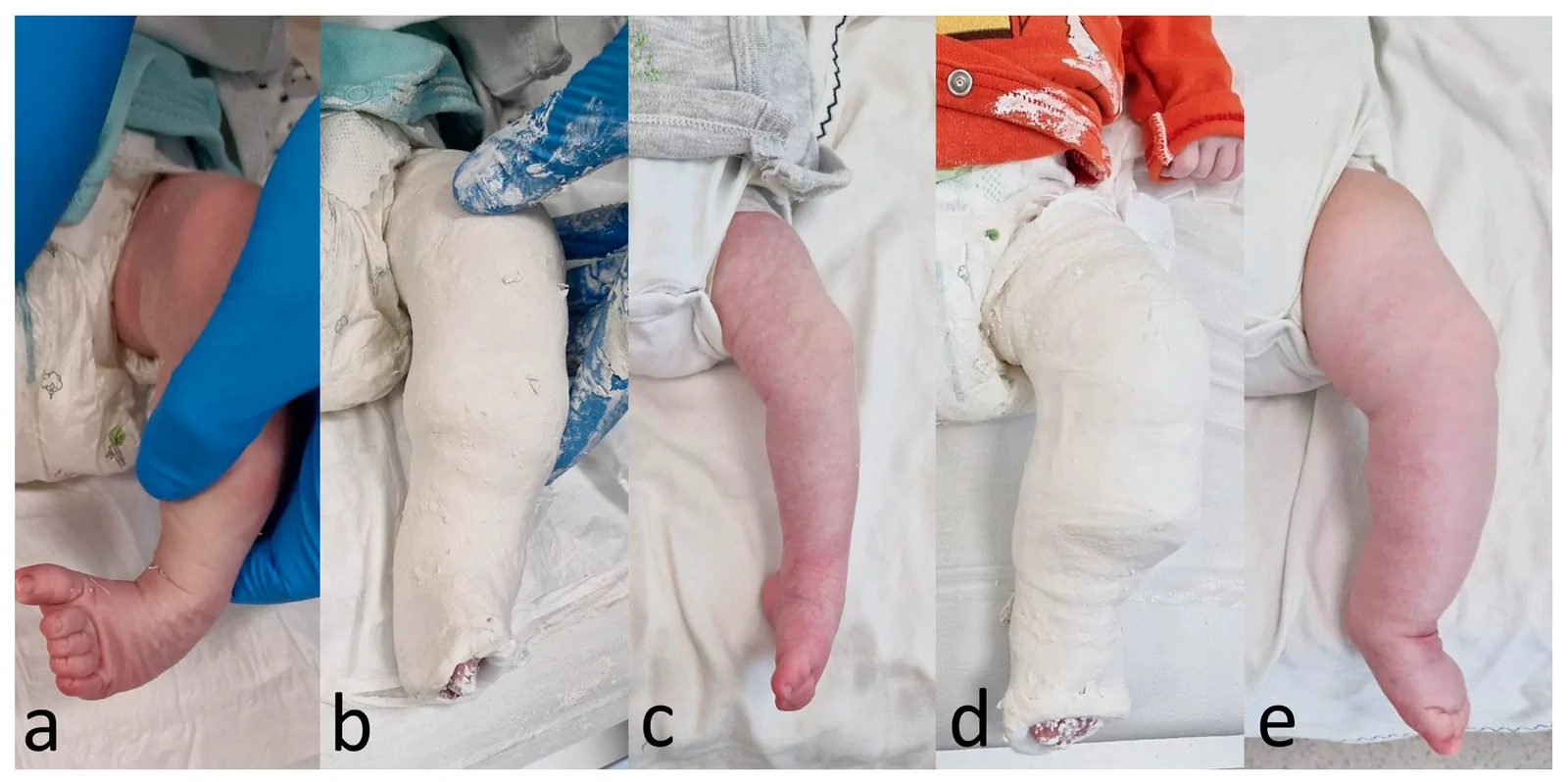
The clinical stages of Ponseti treatment in an infant with idiopathic clubfoot: (**a**) pre-treatment, (**b**) first plaster cast, (**c**) after removal of the plaster cast, (**d**) fourth plaster cast, and (**e**) final correction.

At baseline, the mean Pirani score for the entire cohort was 5.58 ± 0.46 (range, 4–6), and the mean Dimeglio score was 14.98 ± 1.24 (range, 12–17). Baseline Pirani and Dimeglio scores were comparable between Group 1 and Group 2, with no statistically significant differences observed (*p* = 0.654 and *p* = 0.402, respectively) (Table 1). Similarly, no significant differences were identified between groups in final Pirani or Dimeglio scores at the last follow-up (Table 2). A representative clinical course of Ponseti treatment, including the pretreatment appearance, serial casting process, and final correction, is shown in Figure 2.

Percutaneous Achilles tenotomy was performed in all patients prior to the final cast to correct residual equinus deformity. The mean number of casts applied until transition to orthotic treatment was 5.43 ± 0.69 (range, 4–7). The mean number of casts was 5.48 ± 0.77 in Group 1 and 5.38 ± 0.62 in Group 2, with no statistically significant difference between the groups (*p* = 0.536). No significant difference was observed between groups regarding the mean interval between successive casts. However, significant differences were identified in cast removal time, time between cast removal and application of the subsequent cast, and duration of hospital stay (*p* < 0.001 for all comparisons) (Table 2).

**Figure 3:**
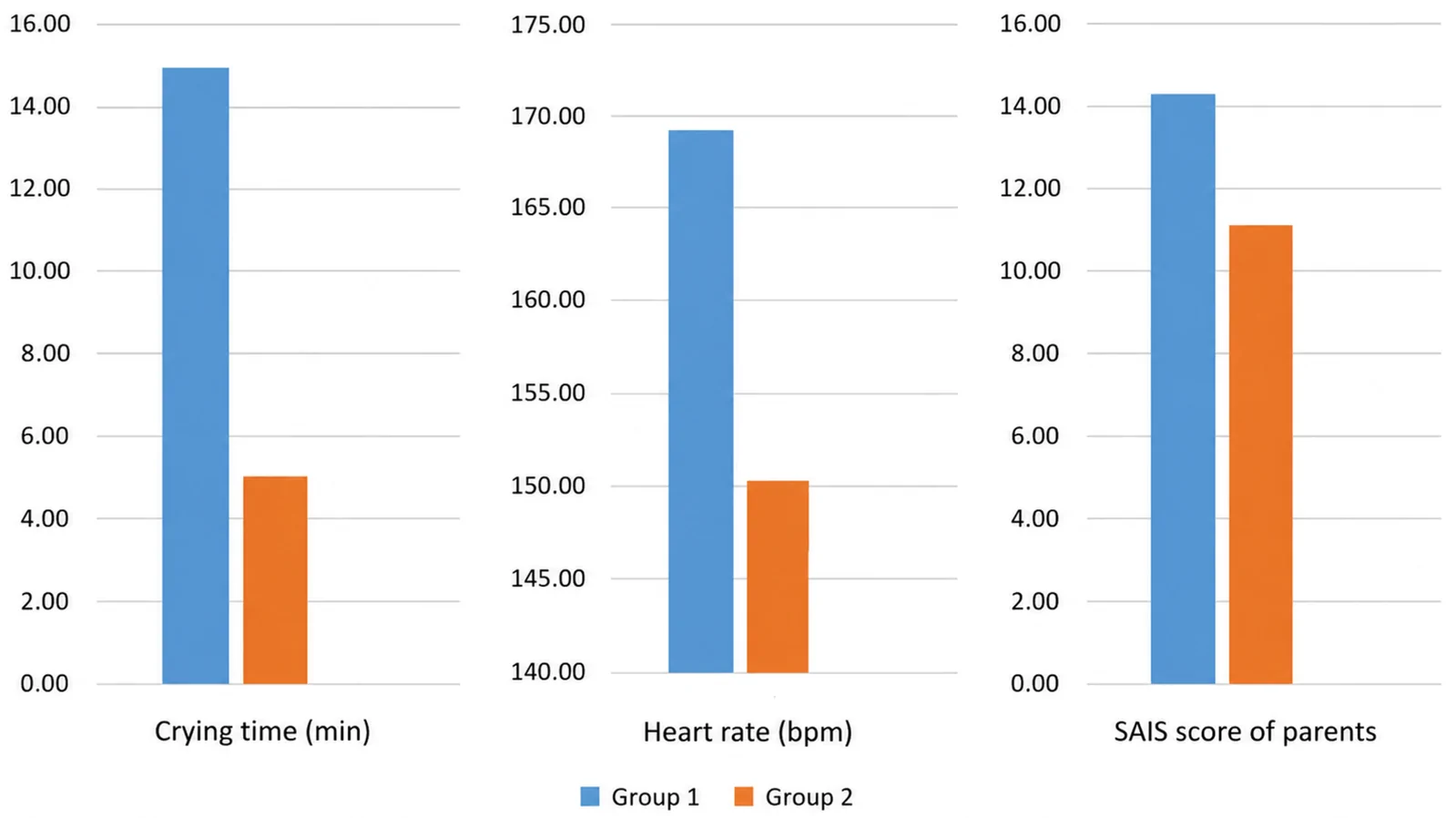
Comparison of anxiety-related parameters (crying time, heart rate, and parental SAIS scores) between Group 1 (oscillating saw) and Group 2 (soaking).

Anxiety-related outcomes differed significantly between groups. The mean crying time during cast removal was 14.91 ± 0.97 min in Group 1 and 5.03 ± 1.59 min in Group 2 (*p* < 0.001) (Figure 3). Similarly, the mean maximum heart rate recorded during cast removal was significantly higher in Group 1 compared with Group 2 (169.02 ± 6.84 vs. 150.55 ± 6.49 beats/min, *p* < 0.001). Parental anxiety scores assessed using the SAIS were also significantly higher in Group 1 than in Group 2 (14.37 ± 3.22 vs. 11.22 ± 2.86, *p* < 0.001).

**Figure 4:**
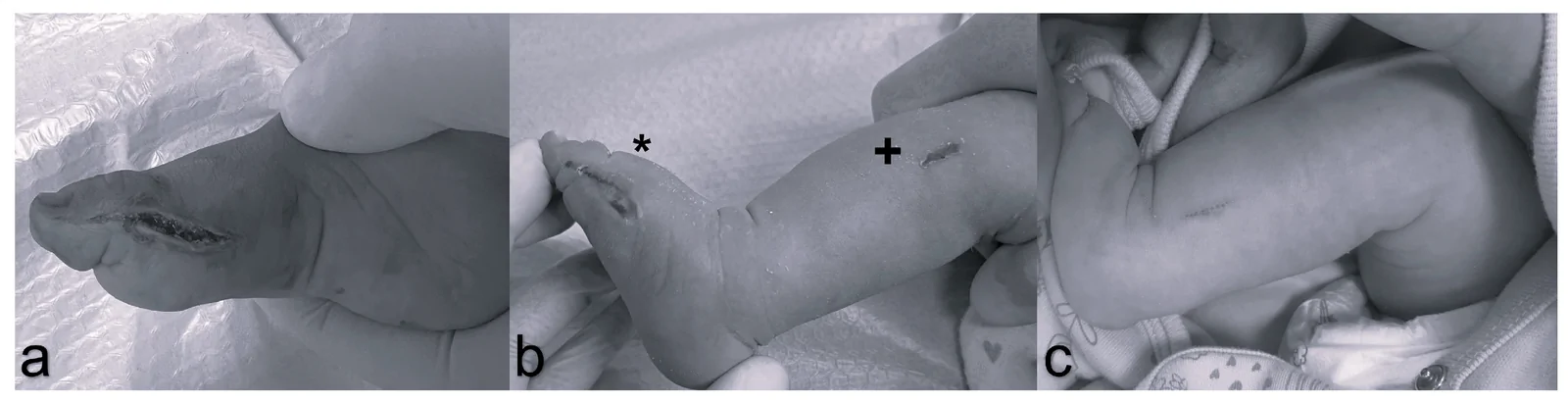
Examples of cast-removal–related skin lesions associated with oscillating saw use: (**a**) full-thickness skin laceration; (**b**) full-thickness skin laceration (*) and second-degree burn in the healing phase (+); (**c**) superficial skin abrasion.

Cast-removal–related complications were observed in 17 patients (20.2%). Of these, 14 patients (82.4%) were in Group 1 and 3 patients (17.6%) were in Group 2, representing a statistically significant difference between groups (*p* = 0.003). The distribution and types of complications are detailed in Table 3 and illustrated in Figure 4.

Exploratory observations revealed interruptions in routine vaccination schedules in nine patients (10.9%), all of whom were in Group 1. No vaccination interruptions were observed in Group 2.

## 4. Discussion

The findings of this study suggest that home cast removal by soaking during the Ponseti procedure may provide clinical outcomes comparable to oscillating saw removal, without increasing the number of casts required for deformity correction. Furthermore, the soaking technique was associated with lower infant distress, reduced parental anxiety, and fewer cast-removal–related complications.

The Ponseti method is well established as an effective treatment for idiopathic clubfoot, with Pirani and Dimeglio scores commonly used to monitor disease severity and treatment success [6,18,19]. Previous studies have emphasized the number of casts required as an indicator of both deformity severity and treatment efficiency [14]. In the present study, the absence of differences in baseline and final clinical scores, as well as in the number of casts between groups, suggests that the method of cast removal does not influence the effectiveness or duration of Ponseti treatment.

The cast removal technique itself was not expected to directly influence the biological effectiveness of the Ponseti method. However, home-based soaking and peeling resulted in a longer interval between cast removal and application of the subsequent cast compared with in-clinic oscillating saw removal. Because prolonged cast-free intervals have been suggested to potentially compromise maintenance of correction during serial casting [14,20], it was important to determine whether this difference affected treatment outcomes. In the present study, despite the significantly longer cast-free interval in the soaking group, no significant differences were observed in the number of casts required or in the final Pirani and Dimeglio scores. These findings suggest that, within the time intervals evaluated in this study, home soaking and peeling was not associated with reduced treatment effectiveness. This observation is consistent with previous reports indicating that modestly prolonged cast-free intervals do not adversely affect correction when appropriate manipulation and casting are subsequently performed [14].

Cast removal using oscillating saws has been associated with procedure-related complications, including skin abrasions and thermal injuries [21,22,23]. While preventive strategies such as safety strips or coated blades have been proposed [21,22,24], their applicability in Ponseti casting is limited due to the specific cast shape and manipulation points required. In this study, cast-removal–related skin complications were significantly more frequent in the oscillating saw group, whereas no serious skin injuries were observed in the soaking group. Although the cast removal procedure in our study was performed by a single experienced technician and protective strips were used during the procedure, the occurrence of cast cut complications justifies the search for alternative cast removal methods, particularly in non-standard procedures such as clubfoot casts.

Manual cast removal using cast shears has also been described as a safe and practical alternative for infant clubfoot casts when performed by experienced healthcare professionals [11]. Unlike oscillating saws, this technique avoids saw-related noise and thermal injury but remains operator-dependent and requires specific technical expertise. Although the utilization of manual cast shears in the Ponseti method mitigates the acoustic distress associated with oscillating saws, it introduces distinct mechanical challenges. Due to the close conformity of Ponseti casts to the contours of the infant’s foot and the fragility of neonatal skin, the high cutting forces sometimes required to divide unevenly softened plaster may increase the risk of iatrogenic skin tears and pressure-related injuries compared with the peeling technique. Because the manual removal with cast shears is not routinely performed at our institution, it was not included as a comparison group in this study. More studies are needed in the future to compare oscillating saws, soaking techniques and manual plaster removal methods.

Anxiety during cast removal is a recognized concern in pediatric orthopedic practice [11,25,26]. Previous studies have demonstrated that noise generated by oscillating saws contributes substantially to procedural anxiety in children and caregivers [11,27]. In the present study, higher infant distress markers and parental anxiety scores were observed in the oscillating saw group, suggesting that soaking-based removal may offer a less stressful alternative for families during Ponseti treatment. However, given the indirect nature of anxiety assessment, these findings should be interpreted with caution. Parental involvement and trust play an important role in pediatric orthopedic care [28,29]. The reduced anxiety and complication rates observed with soaking-based cast removal may support a more family-centered approach to care in appropriately selected patients.

This study has several limitations. First, due to the study design, anxiety-related outcomes in the soaking group were assessed at home and recorded by parents, which may have introduced measurement and recall bias despite standardized instruction. Second, variability in the cast-free interval in the soaking group, related to differences in travel time to the hospital, may have influenced treatment-related parameters. In addition, although a uniform plaster material (POP) was used to enhance procedural consistency, the findings may not be directly generalizable to settings where alternative casting materials such as fiberglass or synthetics are employed. Furthermore, parental educational levels were not recorded in this study. This may affect the correct application of the soaking and peeling technique. Finally, the quasi-randomized allocation process and the inability to blind participants and caregivers represent inherent limitations of the study design. Despite these limitations, the prospective design and standardized treatment protocol strengthen the validity of the findings. Future prospective randomized trials incorporating a standardized assessment of the duration of plaster application and removal, along with parental education levels, appropriate home-based plaster removal methods for different plaster materials, and real-time discomfort and objective physiological stress markers during the procedure could further clarify the relationship between plaster removal and application protocols, the effectiveness of manipulation, and infant comfort.

## 5. Conclusions

In infants treated with the Ponseti method, cast removal using either oscillating saws or soaking techniques resulted in comparable clinical outcomes. However, soaking-based cast removal at home was associated with lower levels of infant distress, reduced parental anxiety, and fewer cast-removal–related complications. These findings suggest that soaking may represent a family-centered alternative for cast removal during Ponseti treatment in appropriately selected patients.

## Figures and Tables

**Table 1:** Baseline characteristics of groups.

	Group 1 (n = 42) (Oscillating Saw)	Group 2 (n = 42) (Soaking)	*p*
**Age (mean ± SD, range) (day)**	6.33 ± 2.52 (2–12)	6.62 ± 2.63 (2–13)	0.613 *
**Sex (n, %)**			**0.827 ** ^†^
Male	27 (60%)	33 (73%)	
Female	18 (40%)	12 (27%)	
**Side (n, %)**			**0.512 ** ^†^
Right	21 (50%)	24 (57%)	
Left	21 (50%)	18 (43%)	
**Dimeglio Score (mean ± SD, range)**	14.86 ± 1.26 (12–17)	15.29 ± 1.23 (12–17)	0.402 *
**Pirani Score (mean ± SD, range)**	5.65 ± 0.39 (4.5–6)	5.50 ± 0.51 (4–6)	0.654 *

**SD**: standard deviation, * Student’s *t*-test, **^†^** Chi-square test, Fisher’s exact test.

**Table 2:** Primary outcomes according to groups.

	Group 1 (n = 42) (Oscillating Saw)	Group 2 (n = 42) (Soaking)	*p* *
**Clinical outcomes**			
Dimeglio score (final)	4.67 ± 0.85 (4–8)	4.57 ± 0.97 (4–9)	**0.765**
Pirani score (final)	2.14 ± 0.56 (1–3.5)	2.11 ± 0.54 (0.5–3)	**0.632**
**Results related to casting**			
Casts	5.48 ± 0.77 (4–7)	5.38 ± 0.62 (4–7)	**0.536**
Cast change intervals (day)	7.06 ± 0.39 (6.3–7.8)	7.13 ± 0.40 (6.4–8)	**0.427**
Time spent for removal cast (min)	5.08 ± 0.85 (3.7–7.4)	18.33 ± 3.02 (12.7–24.5)	**<0.001**
Free time between casts (hour)	1.15 ± 0.28 (0.9–2.1)	4.33 ± 0.35 (4–5.17)	**<0.001**
Time in hospital (hour)	2.14 ± 0.36 (1.54–3)	0.97 ± 0.26 (0.5–1.5)	**<0.001**
**Results of anxiety level assessment criteria**			
Crying time (min)	14.91 ± 0.97 (13.4–17.2)	5.03 ± 1.59 (0.6–8.4)	**<0.001**
Heart rate (beats/min)	169.02 ± 6.84 (153–183)	150.55 ± 6.49 (140–165)	**<0.001**
SAIS score of parents	14.37 ± 3.22 (8.6–21.3)	11.22 ± 2.86 (7.2–18.2)	**<0.001**

Values are presented as the sample mean, standard deviation and range (mean ± SD, range). **SAIS:** Short State Anxiety Inventory Scale, * Student’s *t*-test.

**Table 3:** Frequency of complications related to cast removal techniques.

Complication (n)	Group 1 (Oscillating Saw)	Group 2 (Soaking)
Skin mark/scratch	4	0
Skin laceration (resulting in bleeding)	3	0
Full-thickness skin laceration	2	0
First/second-degree burns	3	0
Redness of skin	2	3
**Total**	**14**	**3**

## Data Availability

The data presented in this study are available on request from the corresponding author due to legal or ethical reasons.

## Abbreviations

The following abbreviations are used in this manuscript:

SAIS	Short State Anxiety Inventory Scale
SPSS	Statistical Package for the Social Sciences
min	Minute
IBM	International Business Machines
